# Uncertainty estimation for temperature measurement with diagnostic ultrasound

**DOI:** 10.1186/s40349-016-0071-x

**Published:** 2016-12-01

**Authors:** Tina A. Fuhrmann, Olga Georg, Julian Haller, Klaus-V. Jenderka, Volker Wilkens

**Affiliations:** 1 Department of Engineering and Natural Sciences, University of Applied Sciences, Eberhard-Leibnitz-Strasse 2, Merseburg, 06217 Germany; 2 Physikalisch-Technische Bundesanstalt, Bundesallee 100, Braunschweig, 38116 Germany

**Keywords:** Ultrasound therapy, HIFU, Dosimetry, Ultrasound thermometry, Accuracy, Uncertainty, Echo-time-shift method, Quality assessment, Safety

## Abstract

**Background:**

Ultrasound therapies are promising, non-invasive applications with potential to significantly improve, e.g. cancer therapies like viro- or immunotherapy or surgical applications. However, a crucial step towards their breakthrough is still missing: affordable and easy-to-handle quality assurance tools for therapy devices and ways to verify treatment planning algorithms. This deficiency limits the safety and comparability of treatments.

**Methods:**

To overcome this deficiency accurate spatial and temporal temperature maps could be used. In this paper, the suitability of temperature calculation based on time-shifts of diagnostic ultrasound backscattered signals (echo-time-shift) is investigated and associated uncertainties are estimated. Different analysis variations were used to calculate the time-shifts: discrete and continuous methods as well as different frames as a reference for temperature calculation (4 s before, 16 s before the frame of interest, base frame). A sigmoid function was fitted and used to calculate temperatures. Two-dimensional temperature maps recorded during and after therapeutic ultrasound sonication were examined. All experiments were performed in agar-graphite phantoms mimicking non-fatty tissue, with high-intensity focused ultrasound being the source of heating.

**Results:**

Continuous methods are more accurate than discrete ones, and uncertainties of calculated temperatures are in general lower, the earlier the reference frame was recorded. Depending on the purpose of the measurement, a compromise has to be made between the following: calculation accuracy (early reference frame), tolerance towards small movements (late reference frame), reproducing large temperature changes or cooling processes (reference frame at a certain point in time), speed of the algorithm (discrete (fast) vs. continuous (slower) shift calculation), and spatial accuracy (interval size for index-shift calculation). Within the range from 20 °C to 44 °C, uncertainties as low as 12.4 % are possible, being mainly due to medium properties.

**Conclusions:**

Temperature measurements using the echo-time-shift method might be useful for validation of treatment plan algorithms. This might also be a comparatively accurate, fast, and affordable method for laboratory and clinical quality assessment. Further research is necessary to improve filter algorithms and to extend this method to multiple foci and the usage of temperature-dependent tissue quantities. We used an analytical approach to investigate the uncertainties of temperature measurement. Different analysis variations are compared to determine temperature distribution and development over time.

## Background

### Ultrasound therapies

Ultrasound therapies have increasingly gained importance over the past few decades [1–4]. They aim to complement or replace standard therapies [5, 6], for instance, chemo- or radiotherapy for cancer treatment [7, 8], thrombolysis of thrombosis [9, 10], or the removal of kidney and gall stones [11, 12]. Additionally, they have evolved for new therapies like viro-, immuno-, or gene therapy [13, 14], drug delivery through the blood-brain barrier [15], or sonophoresis [16–18]. The safety of ultrasound therapies and the accuracy of their treatment plans and of their devices are crucial [19] for the wider acceptance and usage of the new therapies, for patient safety, and for administrative approval.

### Safety assurance before the treatment

Considerable effort has been made towards objective safety criteria during treatment in the past few years, for example, based on the mechanical and thermal index [20], the maximum allowed temperature on the transducer surface [21], or real-time temperature control.

There is a lack of standards for implementation and execution of quality assurance processes before the therapy or a single treatment session. This is partially because there are not many possibilities to easily, but at the same time reliably, e.g. validate the algorithms of a treatment planning programme, calculated doses, and estimated side effects or to test therapy devices before treatment in medical facilities [22]. It is a major shortcoming for the reproducibility of treatment sessions and the comparability of clinical studies [23]. Tools needed for the tasks mentioned above must be easy-to-handle, reproducible, accurate, reliable, and inexpensive.

One possibility to test the functionality and the constancy of therapeutic ultrasound devices could be to sonicate a tissue-like phantom with standardized parameters and to monitor temperatures in a region of interest during a certain time (e.g. only during or during and after sonication). In this study, only non-fatty tissue phantoms are used to investigate the general feasibility of our methods.

### Temperature measurement

Temperature measurement with diagnostic ultrasound is appropriate since it is relatively cheap, easily accessible in medical facilities, capable of use in real time, and allows two- or three-dimensional imaging. It thus has major advantages when being used for quality assessment compared to magnetic resonance or thermocouple measurements.

The idea of using ultrasound measurement for non-invasive thermometry has been promoted already 20 years ago [24, 25] and has been successively improved since then. Progress was made i.a. in the fields of two-dimensional [26], three-dimensional [27], and compound imaging [28], improved algorithms [29–33], and the estimation of limitations [34]. Furthermore, the applicability of the method in different media was described: homogeneous [35, 36] and multilayer [30] phantoms, and in tissue in vitro [37–40] and in vivo [41]. A comprehensive review can be found in [42].

### What to expect in this paper

In our study, we investigated the suitability and uncertainties of a common method for temperature measurement: the echo-time-shift method. Different analysis variations were used to calculate the time-shifts: discrete and continuous algorithms with the reference frame for each frame being either the frame 4 or 16 s before the frame of interest or the base frame. Moreover, we introduce an uncertainty calculation based on mathematical analysis and show specific possibilities to decrease uncertainties.

## Methods

### A. Temperature calculation with echo-time-shift and echo-index-shift

The basic physical concepts used for measuring temperatures with ultrasound are the thermal expansion of the heated medium and the change of its speed of sound. Both effects result in a time-shift of the backscattered ultrasound signal. The speed of sound is usually dependent on temperature one-to-one up to approximately 45 °C in water-based media, including non-fatty tissue [43], and most tissue-mimicking materials. In human tissue, the absolute value of the speed of sound and its dependence on temperature are strongly related to the particular tissue composition. Therefore, no absolute temperatures can be measured with ultrasound, but rather temperature changes *δ𝜗* to a reference frame, as shown in detail by Simon et al. [26]:

1 $$  \delta\vartheta =k \cdot \frac{c(z,\vartheta_{0})}{2} \cdot \frac{\partial}{\partial{z}} \left(\delta t(z)\right)  $$

with

2 $$ k=\frac{1}{\alpha-\beta}   $$

Here, *c* is the speed of sound in the medium at the initial frame at constant initial temperature *𝜗*_0_, $\frac {\partial }{\partial z}$ is the spatial derivation along axial depth *z* (along a scanline), and *δt* is the time-shift of the backscattered signal. *c* is assumed to change approximately linearly with temperature with the thermal coefficient of the speed of sound $\beta = \frac {1}{c(\vartheta _{0})} \frac {\partial c(\vartheta)}{\partial \vartheta }$. *α* is the linear coefficient of thermal expansion. For our purposes, *α* and *β* are both considered to be independent of temperature and of axial depth, because a homogeneous phantom is used. Therefore, they can be combined to a value *k* (Eq. 2).

*k* is the proportionality factor between temperature change on the one hand, and relative change of both speed of sound and length on the other hand. In other words, it specifies the proportionality between temperature change and the shift of the signal, as shown later. *k* is larger than zero for non-fatty tissue and smaller than zero for fatty tissue. Thus, major problems will occur if the diagnostic ultrasound scanline passes fatty and non-fatty tissue and if a single value is presumed for *k* in these cases. In our study, we focus on a non-fatty tissue phantom. Prospects for the application in inhomogeneous phantoms, for instance, by using a depth-dependent *k* and an iterative calculation procedure, must be investigated in further studies. *k* only depends on the particular material but neither on temperature nor on depth.

The measured RF signal is a discrete time series. It is recorded with a sampling frequency *f*_sample_ at equidistant time steps. To consider these circumstances, Eq. 1 is rearranged using index *i* from each measured data point along a scanline, beginning with index 1 at the beginning of the phantom:

3 $$\begin{array}{*{20}l} \delta\vartheta &=k\cdot\frac{c(z,\vartheta_{0})}{2}\cdot \frac{\partial \left(\frac{\delta i}{f_{\text{sample}}}\right)}{\partial \left(\frac{i}{f_{\text{sample}}}\cdot \frac{c(z,\vartheta_{0})}{2}\right)} \end{array} $$

4 $$\begin{array}{*{20}l}  &=k\cdot\frac{\partial}{\partial i}(\delta i) \end{array} $$

Here, *δi* accounts for the incremental index-shift, where incremental means additive along axial depth, and $\frac {\partial }{\partial i}(\delta i)$ is the index-shift. Note that *δt* and *δi* are referred to as the incremental time-shift and the incremental index-shift, respectively, since they add up along a scanline. Their spatial derivation is then solely called time- or index-shift.

For a given experiment, the absolute values of *δi* depend on the sampling frequency and interpolation of the original backscattered RF signal. The incremental index-shift is calculated with cross-correlation as explained in more detail in the “D. Incremental index-shift calculation methods” section.

### B. Temperature calculation with a sigmoid function fit

During high-intensity focused ultrasound (HIFU) sonication, only a small volume is heated. Therefore, the incremental index-shift profile along a scanline crossing the heated area is as follows (in a homogeneous phantom): It is constant (usually zero) in front of as well as behind (usually non-zero) the heated zone and rises along it. Hence, a sigmoid function can be used to describe the data as proposed in [31]:

5 $$  \delta i = \frac{a}{1+e^{-b(i-c)}}+d  $$

The fit lowers the impact of noise which is due to the decorrelation of RF data on the calculated incremental index-shifts. The noise occurs especially in and axially behind the heated zone. The spatial differentiation is

6 $$  \frac{\partial}{\partial i} (\delta i) = ab \frac{e^{-b(i-c)}}{\left(1+e^{-b(i-c)}\right)^{2}},  $$

where *i* is the index of the data point of the pre-processed (i.e. interpolated or frequency filtered) RF signal, *e* is Euler’s number, and *a* to *d* are fit parameters. *d* accounts for the incremental index-shift that arose on that scanline axially before the beginning of the phantom, *a* is the maximum incremental index-shift difference to *d*, *b* is the slope at the turning point and therefore determines the value of maximum temperature, and *c* is the location of the turning point and therefore the location of maximum temperature.

A sigmoid fit was performed on every scanline. The fit parameter *b* (in index-shifts per index) is much smaller than one and having a maximum of 0.01 in our calculation during the strong temperature rise in the focus zone. Therefore, it was limited to ±0.015 for fitting. As mentioned before, *d* is usually zero. With the fit parameters *a* to *d*, the temperature was calculated as follows (Eq. 6 into Eq. 4):

7 $$  \delta \vartheta = kab \frac{e^{-b(i-c)}}{\left(1+e^{-b(i-c)}\right)^{2}}  $$

### C. Uncertainties of calculated temperatures

The uncertainty analysis was performed according to the *Guide to the Expression of Uncertainty in Measurement* [44]. Overall uncertainty *u*_*f*_ for a value *f* (in our case, the calculated temperature values) is obtained via the Gaussian propagation of uncertainties

8 $$  u_{f} = \sqrt{\sum\limits_{n=1}^{N}\left(\frac{\partial f}{\partial x_{n}} u_{n}\right)^{2}},  $$

where *x*_*n*_ are the independent variables (*k*, fit parameters *a*, *b*, and *c*), *N* is their number, and *u*_*n*_ is the uncertainty of the associated variable *x*_*n*_.

#### Uncertainty of *k*

Multiple measurements were performed explicitly for determining the value of *k*, using the transformation of Eq. 4:

9 $$  k = \frac{\vartheta}{\frac{\partial}{\partial i}(\delta i)}  $$

The uncertainty of *k* is determined twice, in each case considering different influences. Firstly, the standard deviation of all values of *k* was calculated and, secondly, the Gaussian uncertainty (derived from Eq. 8):

10 $$\begin{array}{*{20}l}  \frac{u_{k}}{k} &= \sqrt{\left(\frac{u(\delta \vartheta)}{\delta\vartheta}\right)^{2}+ \left(\frac{u\left(\frac{\partial }{\partial i}\delta i\right)}{\frac{\partial }{\partial i}\delta i }\right)^{2}} \end{array} $$

A linear fit was used for the incremental index-shift data where the slope *m* corresponds to $\frac {\partial }{\partial i}(\delta i)$. Therefore, Eq. 10 is simplified as

11 $$  \frac{u_{k}}{k} = \sqrt{\left(\frac{u(\delta \vartheta)}{\delta\vartheta}\right)^{2}+ \left(\frac{u(m)}{m}\right)^{2}}  $$

#### Uncertainty of temperature

Uncertainties of calculated temperatures were derived by using the residuals and Jacobian matrix of the sigmoid fit. The uncertainty contributions of the parameters $\frac {\partial \vartheta }{\partial x_{n}}$ were calculated using Eq. 7, substituting *r*=−*b*(*i*−*c*) for better readability:

12 $$\begin{array}{*{20}l}  \frac{\partial}{\partial k}(\delta\vartheta) &=\frac{\delta\vartheta}{k} \end{array} $$

13 $$\begin{array}{*{20}l} \frac{\partial}{\partial a}(\delta\vartheta) &=\frac{\delta\vartheta}{a} \end{array} $$

14 $$\begin{array}{*{20}l} \frac{\partial}{\partial b}(\delta\vartheta) &=\frac{\delta\vartheta}{b}\left[ 1-r\frac{1-e^{r}}{1+e^{r}} \right] \end{array} $$

15 $$\begin{array}{*{20}l} \frac{\partial}{\partial c}(\delta\vartheta) &=\delta\vartheta b^{2} \frac{1-e^{r}}{1+e^{r}} \end{array} $$

**Fig. 1:**
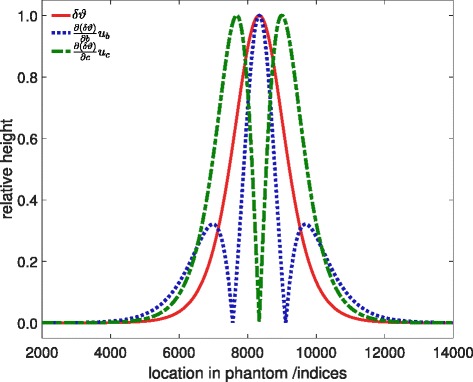
Uncertainty contributions of fit parameters *b* and *c*. For reasons of spatial comparison, the ideal temperature curve is shown, too

The relative contributions of *b* (Eq. 14) and *c* (Eq. 15) are shown in Fig. 1. The contribution of *b* has its maximum at the location of the therapeutic ultrasound focus and quickly decreases to zero with two smaller side lobes. The uncertainty contribution of *c* is negligible in the focus but has two large side lobes at the steep slope of the temperature curve. To some extent, it includes the spatial uncertainty in the calculation, but not completely (i.e. not uncertainties that might be due to interval size). Even though *b* ^2^ is much smaller than one (*b* is arbitrarily limited to ±0.015), it cannot be neglected in Eq. 15 since *u*_*c*_ can become very large. To obtain smaller uncertainty values of *b*, this fit parameter could be estimated more restrictively. The fit parameter *d* is not relevant for temperature calculation and therefore also not for uncertainty calculation.

The fit parameters are not independent on each other but their dependency is not known in detail. This correlation could be examined in the future for instance with Monte Carlo simulations, also taking interval sizes into account. Therefore, the uncertainties due to fit parameters add to a general fit uncertainty budget without squaring.

The uncertainty budget of a calculated temperature value can now be estimated to the following:

16 $$ \begin{aligned} \frac{u_{\delta\vartheta}}{\delta\vartheta}= \sqrt{ \left(\frac{u_{k}}{k}\right)^{2}+ \sum_{\text{ref}}\left(\frac{u_{a}}{|a|}+ \frac{u_{b}}{|b|}\left[ 1-r\frac{1-e^{r}}{1+e^{r}}\right]+ u_{c} b^{2} \frac{1-e^{r}}{1+e^{r}}\right)^{2} }, \end{aligned}  $$

where the subscript “ref” means the sum over the associated reference frames. If the reference frame is another than the initial frame, uncertainties due to fitting add up and can only stay the same or grow larger. Otherwise, they can also decrease over time since they are independent of any calculation before. A decrease during cooling is possible, because at lower temperatures, less decorrelation due to spatial extension and fewer losses of interferences occur. This leads to less noise and a more suitable fit. The method of calculating uncertainty presented here assumes that incremental index-shifts can be measured continuously and filtered reasonably.

### D. Incremental index-shift calculation methods

**Fig. 2:**
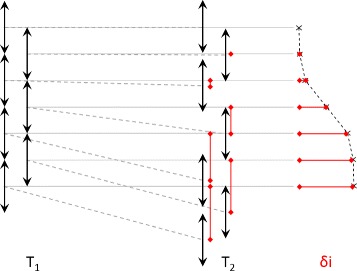
Schematic diagram of cross-correlation leading to incremental index-shifts *δi*. The RF signal was recorded at different wall-clock times *T*. One frame consists of multiple scanlines that were subdivided in half-overlapping intervals of six wavelengths along the axial direction (*left*). Cross-correlation was calculated between the same axial intervals on the same scanlines recorded at different times (*middle*) resulting in the incremental index-shifts (*right*)

Incremental index-shifts *δi* were used for temperature calculation and could be transformed into incremental time-shifts by dividing them through the sampling frequency and an interpolation factor applied on the original RF data. The shifts were determined with cross-correlation by comparing the same axial intervals of the backscattered ultrasound signal of two different frames with each other, one after another (Fig. 2). The interval length is six wavelengths (0.912 mm in our measurements).

#### Discrete cross-correlation

Whole-numbered cumulative index-shifts *δi* were calculated with discrete cross-correlation. *δi* lays within the interval [ −*I*+1, *I*−1], with *I* being an odd axial interval length in indices for simplicity. For discrete backscattered RF signals *s*_1_ and *s*_2_ at wall-clock times *T*_1_ and *T*_2_, the cross-correlation sequence is

17 $$ c(i;q)=\frac{1}{N} \sum\limits_{i^{\prime}=-\frac{I-1}{2}}^{\frac{I-1}{2}} s_{1}(i+i^{\prime})\cdot s_{2}(i+i^{\prime}+q)  $$

with *N* being a normalization factor and *q* being the index of the cross-correlation sequence. The incremental index-shift *δi* is the index *q* of the maximum of the cross-correlation sequence

18 $$ \delta i_{d}(i) =\text{index}\left(\max \left(c\left(i;q\right) \right) \right)  $$

#### Continuous cross-correlation

Real-valued index-shifts can be obtained using a continuous cross-correlation algorithm. For this, a complex analytic RF signal $\hat s(i)=s(i)+j\tilde {s}(i)$ was used, where *j* is the imaginary unit. The imaginary part was calculated with a Hilbert transform. The index-shift was first calculated as shown before (discrete). The interval of the reference frame was then kept constant and intervals at the frame of interest were shifted about the discrete shift. Then, a real-valued, continuous incremental index-shift *δi*_*c*_ was calculated. In this work, a method similar to the one described by Loupas [45] and altered by Simon [26] is used

19 $$ c(i;q)=\frac{1}{N} \sum\limits_{i^{\prime}=-\frac{I-1}{2}}^{\frac{I-1}{2}} \hat{s}_{1}(i+i^{\prime}) \cdot \hat{s}_{2}^{*}(i+i^{\prime}+q),  $$

where ^∗^ denotes the complex conjugate. The cross-correlation sequence *c* is complex. The zero crossing of the angle of *c* is the phase-shift (in radians) that is transformed into the continuous index-shift by multiplying it with a conversion factor *δi*_*cf*_ $\left (\delta i_{cf}=\frac {f_{\text {sample}}}{\pi f_{\text {transducer}}}\right)$. The continuous index-shift can be calculated as follows [26]:

20 $$  \delta i_{\mathrm{c}}(i)=\frac{2 \varangle c(i;0)}{\varangle c(i;1)-\varangle c(i;-1)} \,\, \delta i_{\text{cf}}  $$

Here, ∢ denotes the four-quadrant inverse tangent (*atan2* in many programming languages). Continuous phase-shifts are detectable within ±*π* and therefore the index-shifts *δi*_*c*_ within ±*δi*_cf_ *π*. The intervals were truncated to [ −1.5, 1.5] because discrete incremental index-shifts were calculated before, so that |*δi*_*c*_| should be smaller than or equal to 1.0.

For implementation, of both cross-correlation calculations, the MATLAB function *xcorr* was used. Both, discrete and continuous incremental index-shifts, were added up to obtain the final incremental index-shift:

21 $$  \delta i(i)=\delta i_{\mathrm{d}}(i)+\delta i_{\mathrm{c}}(i)  $$

#### Extension to more dimensions

The algorithm could simply be changed for a three-dimensional cross-correlation calculation:

22 $$ \begin{aligned} &c(i,l,m;q,t,v)=\frac{1}{N} \sum\limits_{i^{\prime}=-\frac{I-1}{2}}^{\frac{I-1}{2}} \sum\limits_{l^{\prime}=-\frac{L-1}{2}}^{\frac{L-1}{2}} \sum\limits_{m^{\prime}=-\frac{M-1}{2}}^{\frac{M-1}{2}} \\ &\hat{s}_{1}(i+i^{\prime},l+l^{\prime},m+m^{\prime}) \cdot \hat{s}_{2}^{*}(i+i^{\prime}+q;l+l^{\prime}+t,m+m^{\prime}+v)  \end{aligned}  $$

Here, *I*, *L*, and *M* are the odd axial interval length as well as odd numbers of lateral and elevative scanlines. The distance between the lateral and elevative scanlines should be approximately equal. The function values of *c* at lags *t*=0 (lateral) and *v*=0 (elevative) are used:

23 $$ \delta i_{\mathrm{c}}(i,l,m)=\frac{2 \varangle c(i,l,m;0,0,0)}{\varangle c(i,l,m;1,0,0)-\varangle c(i,l,m;-1,0,0)}  $$

#### Applied calculation variations

Some properties can be modified when performing the calculations. These are the reference frame for and the level of continuity of the incremental index-shift calculation as well as the number of scanlines used for the cross-correlation calculation. All three influence the actual value of the incremental index-shift and its uncertainty but should finally result in the same calculated temperatures.

**Fig. 3:**
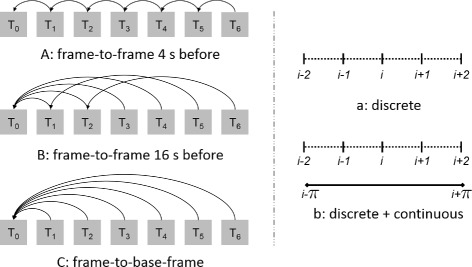
Calculation methods for incremental index-shifts. All six combinations of reference frames (**a**–**c**) and levels of continuity (**a**, **b**) were investigated

As reference frames (Fig. 3, left), the previous frame (4 s before the current frame, A), the fourth frame before it (16 s before, B), and the base frame (C) were used. These settings account for clinical usage with small mechanical movements (A), for laboratory conditions without any movements (C) and an intermediate setting (B). For all methods, discrete (a) and continuous (b) calculations were performed to obtain incremental index-shifts. For reasons explained later, uncertainties were only calculated for the continuous methods.

For the comparison of incremental index-shift calculation variations, the same RF data was used. The RF signal was linearly interpolated from a 40 MHz sampling frequency to a 160 MHz equivalent to obtain smaller time steps between consecutive values in the backscattered ultrasound signal and therefore larger incremental index-shifts. This procedure is especially necessary for discrete methods.

### E. Research design

#### Measurement setup

**Fig. 4:**
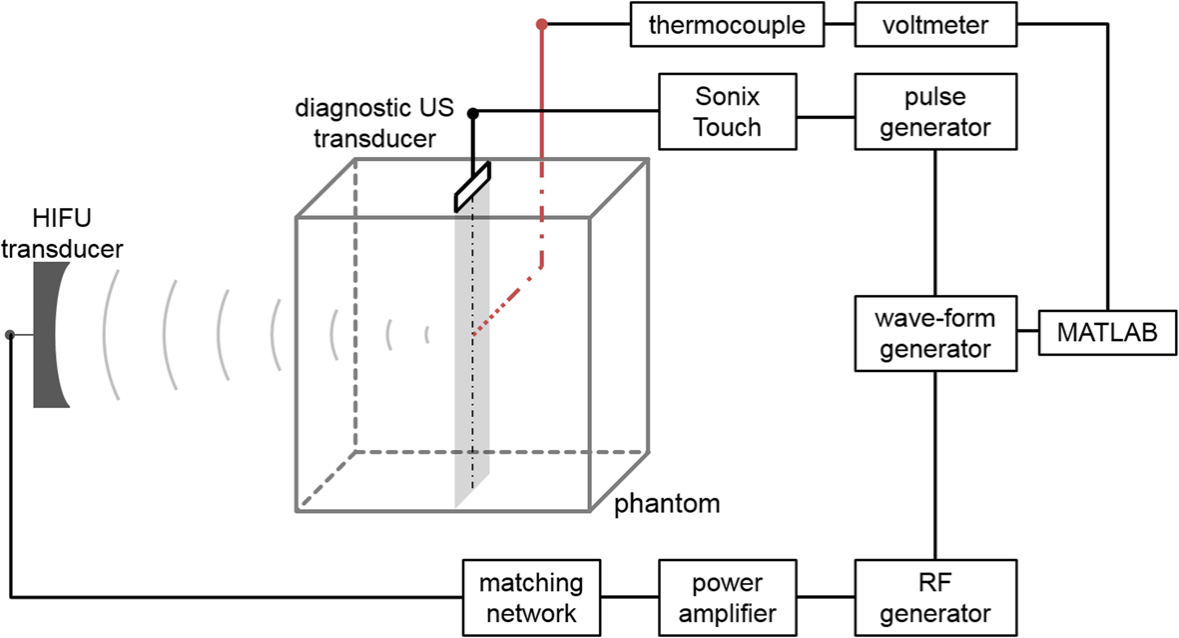
Control scheme. Experimental setup for measuring temperatures during and after HIFU sonication

In Fig. 4, the experimental setup is shown for temperature measurements. A HIFU transducer (model H-102, Sonic Concepts, 1.1 MHz centre frequency, 51.74 mm focal depth, 64.00 mm diameter) and a diagnostic ultrasound transducer (SonixTOUCH, Analogic Corporation, probe L14-5/38, operated at 10 MHz, 40 MHz sampling frequency) were arranged outside the phantom as shown. The thermocouple tip (TCdirect, type K, 0.5 mm outer casing) was placed in the focus zone of the HIFU transducer and within the imaging plane of the diagnostic ultrasound. Experiments were performed with a cubic agar-graphite phantom similar to the one in [46] (10 cm edge length, recipe: 850 ml water, 25.5 g agar-agar, 127.5 ml isopropyl, 10.0 g graphite), and in a large water tank filled with degassed and deionized water. The temperature of the water was measured with a resistance thermometer (Hettstedt GmbH, Pt100). The phantom was placed on a mount above an ultrasound absorber. Before measurement, the phantom and water were at the same baseline temperature at approximately 20 °C. 20 °C is a common room temperature in laboratories and clinics, where the quality measurements will be performed. Body temperature is not necessary since we are not referring to methods being used in humans later on. The experimental process as well as data acquisition was controlled by MATLAB (R2015b).

#### Procedure of temperature measurement

At the beginning, multiple frames at baseline temperature were recorded. Therapeutic ultrasound was run in discontinuous mode (3.85 s on, 150 ms off, duty cycle 96.25 %) for 80 s and actively heated the phantom. The ultrasound power was 20.8 W, as determined from radiation force balance measurements with the same sonication conditions. In every break (every 4 s), two diagnostic ultrasound frames were recorded. Afterwards, the phantom was allowed to cool down but it was not actively cooled. Diagnostic ultrasound frames were recorded for approximately another 2 min every 4 s. It turned out that the signal quality remains the same in both frames recorded during one HIFU break and therefore acquiring one frame in each break would be sufficient. A larger duty cycle could be implemented. Only one frame of each measuring break was used for the calculations. For temperature analysis, MATLAB was used (*lsqcurvefit* for sigmoid fitting with lower and upper boundaries and 4000 iterations per fit, *nlparci* to obtain the 95 % confidence intervals of the fit parameters with use of the residuals and Jacobian matrix).

#### Procedure of measurement of k

The value *k* was obtained by actively heating and cooling the water to well-defined temperatures (20 °C to 44 °C in steps of 8 °C) and waiting for the phantom to adjust its temperature. A smaller water bath, a single transducer (5 MHz, V309, 5.0/0.5 170680, Panametrics), and a pulser/receiver (US-Key, Lecoeur Electronique) were used instead of the linear array that was used for HIFU measurements. This was due to the long exposure time to high water temperatures and to prevent damage to the linear array. To improve the measurement of *k*, a smaller phantom and a linear transducer could be used. Smaller temperature steps and multiple scanlines could then be analysed. Because of the long measurement time, the phantom was put into a standard freezer plastic bag to improve the temporal stability of the chemical composition, mainly the isopropanol content. The temperature measuring devices (thermocouple and resistance thermometer) were calibrated at PTB. Before the experiments were performed, manufacturers’ data on the size of the focus zone of the HIFU transducer were re-checked with hydrophone measurements in water.

### F. Algorithm

#### General proceeding

Algorithms for calculating *k* and temperature rises are very similar. The implementation of the algorithms used is not suitable for real-time measurement since this is not necessary under laboratory conditions. It could simply be changed, e.g. by calculating temperatures immediately after acquiring the diagnostic ultrasound frames, by using parallel programming, or MATLAB functions that calculate in parallel. The procedure was as follows (MATLAB commands in italics and in parentheses):

1. Acquire experimental data including a baseline image at homogeneous temperature distribution
2. Signal pre-processing
  Subtract any existing offset
  Interpolate RF signal to a 160 MHz equivalent sampling frequency (*interp1*)
  Calculate Hilbert transform (*hilbert*)
  Calculation of *k*: sort measurements from low (baseline) to high temperature
3. Calculate discrete incremental index-shifts (*xcorr*)
4. Calculate continuous incremental index-shift (*xcorr*, *angle*)
5. Filter incremental index-shifts along the axial direction for every scanline (*medfilt1*)
6. Linear fit (*polyfit*) for calculation of *k* or sigmoid fit (*lsqcurvefit*, *nlparci* for uncertainties) for temperature calculation
7. Calculate *k* (Eq. 9) or temperatures (Eq. 7) and uncertainties (Eqs. 11 and 16)
8. Add temperatures and uncertainties (without uncertainty due to *k*) to those from reference frames
9. In case of temperature calculation, add uncertainty of *k*

The index *i* was counted in relation to the front wall of the phantom to meet the requirements of the method, where the phantom has to expand away from the transducer. Half-overlapping intervals were used for cross-correlation calculations.

#### Filtering of index-shifts

Filtering of the calculated incremental index-shifts is essential for this method because outliers strongly influence the quality of both, linear fits for the calculation of *k* and sigmoid fits for temperature evaluation, including the uncertainties of the fit parameters. For temperature calculation, a median filter was applied twice on incremental index-shifts. First, five neighbours (eleven values) were taken into account, then one neighbour (three values).

A better but very challenging option would be a filter algorithm only based on the incremental index-shift difference between axially consecutive incremental index-shifts. If the difference is larger than an allowed value, the axially consecutive incremental index-shift is defined to be an outlier. The allowed difference is dependent on the therapeutic ultrasound power and absorption coefficient of the medium, the size of the focus zone, the degree of interpolation of the RF sampled data, the time within the heating or cooling process, and the reference frame used. We verified the general possibility of such a filter algorithm and obtained good results. However, we did not use it here because of its difficult implementation and up to now unmanageable usage in a clinic. It might nevertheless be useful for constancy testing purposes, where the above-mentioned quantities are usually known.

In this study, neither spatial nor temporal filtering was applied to the final temperature maps since the filtering influences uncertainty of the methods in an incalculable way. For validation of treatment planning algorithms or laboratory dosimetry, temperature filtering is possible and recommended. As shown later, our results suggest that filtering of temperature maps could improve overall accuracy and reliability of single calculated temperature values.

## Results and discussion

### A. Final values and uncertainties of *k*

Properties of the phantom material are introduced into temperature calculation via the material dependent value *k* (Eq. 2).

**Fig. 5:**
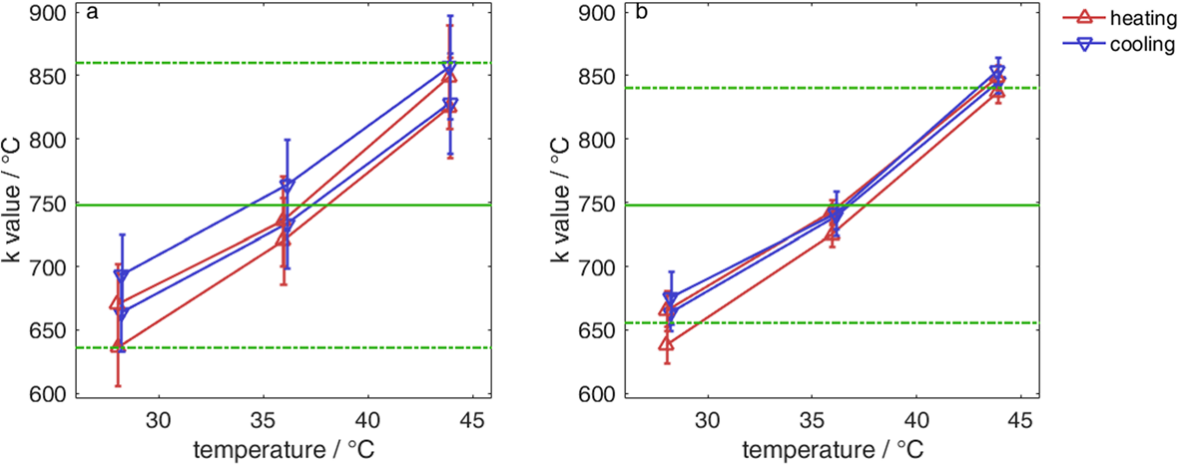
Values of *k* for a phantom with a plastic coating. *k* is for discrete frame-to-frame calculations (**a**) (748.0±112.0 °C) (uncertainty 15.0 %) and (747.9±92.4 °C) (uncertainty 12.4 %) for continuous frame-to-base-frame calculations (**b**), respectively

*k* was found to be (749.9±92.4 °C). The best achievable uncertainties for *k* were 12.4 % when using a continuous frame-to-base algorithm and 15.0 % when using a discrete frame-to-frame algorithm (Fig. 5 and Table 1). The final values of *k* for the different computation methods were obtained by averaging the values in the range 20 to 44 °C. For the determination of *k*, only a discrete frame-to-frame algorithm was compared to a continuous frame-to-base algorithm. More outliers were present in the calculated incremental index-shift data of the frame-to-base algorithm which is due to larger temperature differences and the bad signal-to-noise ratio of the backscattered ultrasound signal in this measurement setup, especially in larger axial depth.

**Table 1:** Values and uncertainties of *k*

	Final *k*	Standard deviation	Gaussian uncertainty	Uncertainty
Discrete frame-to-frame	748.0 °C	76.6 °C (10.2 %)	35.4 °C (4.8 %)	112.0 °C (15.0 %)
Continuous frame-to-base	747.9 °C	80.1 °C (10.7 %)	12.3 °C (1.7 %)	92.4 °C (12.4 %)

Standard deviation was calculated between all *k* in the measurement, Gaussian uncertainties of the values of *k* were averaged, and (final) uncertainty was obtained by adding both uncertainties

*k* was much more dependent on temperature than previously reported in the literature (e.g. [26]) and also than the widespread application of the echo-time-shift method would suggest. It is not constant within the considered temperature range as is assumed for the method. This is due to the non-linear dependence of the speed of sound on the temperature which strongly influences the standard deviation. Therefore, the standard deviation of all calculated values of *k* plus the uncertainty due to the measurement (devices) and the calculation (algorithm including filtering and fitting) were combined to the final uncertainty of *k*. Ideally, both would be of the same size and due to the same physical and algorithmic effects.

The independence of *k* on temperature is inherent to non-fatty tissue [43] and therefore a necessary property of phantoms mimicking non-fatty tissue. Up to 44 °C, *k* can be considered temperature independent with the uncertainties shown in Table 1. Then, small temperature changes are overestimated and large ones underestimated. Depending on the usage of the method, this must be taken into account and possibly be corrected. The temperature dependence, especially at temperatures higher than 44 °C, is not a fundamental limitation for laboratory dosimetry because it is generally possible to implement smaller baseline temperatures. Continuing research should investigate as to what extent results are transferable to higher temperatures. The temperature dependence of *k* might be a strong limitation for clinical applications [34] because of the higher initial temperatures (36 °C).

To further decrease uncertainty due to the calculation of *k*, calculations could be optimized, for instance, by using better filter algorithms for outlying incremental index-shifts or by using transducer arrays that allow performing more comprehensive statistics on the calculation of *k*.

### B. Temperature changes: comparison of different calculation methods

To compare different calculation variations and their uncertainties, in this part, we analyse the (1) incremental index-shifts, (2) temperature-time curves, and (3) temperature maps.

#### 1a. Discrete vs. continuous incremental index-shifts

**Fig. 6:**
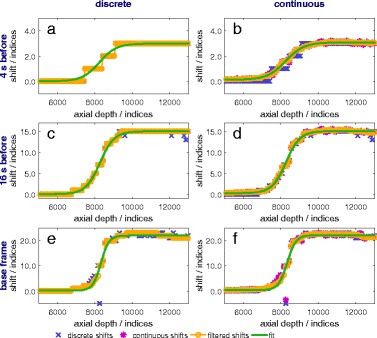
Incremental index-shifts after 20 s of heating. Shifts are shown for discrete (**a**, **c**, **e**) and continuous (**b**, **d**, **f**) methods using reference frames 4 and 16 s before the frame of interest as well as the base frame

**Fig. 7:**
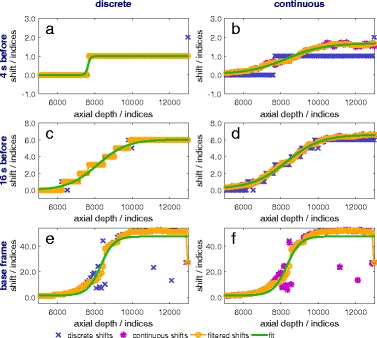
Incremental index-shifts after 80 s of heating. Shifts are shown for discrete (**a**, **c**, **e**) and continuous (**b**, **d**, **f**) methods using reference frames 4 and 16 s before the frame of interest as well as the base frame

**Fig. 8:**
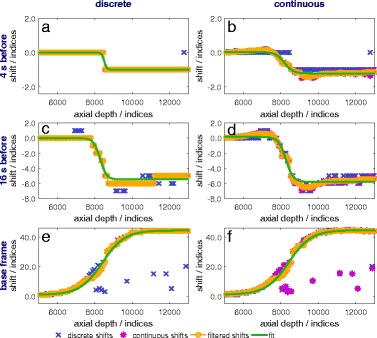
Incremental index-shifts after 100 s (80 s of heating and 20 s of cooling). Shifts are shown for discrete (**a**, **c**, **e**) and continuous (**b**, **d**, **f**) methods using reference frames 4 and 16 s before the frame of interest as well as the base frame

In Figs. 6, 7, and 8, the discrete, continuous, in case of the continuous methods, and the filtered incremental index-shifts as well as the calculated sigmoid function fit are shown for all calculation variations. More noise is present in the calculated shifts axially behind the location of the maximum temperature and behind the thermocouple. The first case is due to the thermoacoustic lens effect [26] and the second to the reflection at the surface of and absorption within the thermocouple. Accurate detection and filtering of incremental index-shifts are essential for an accurate fit and low uncertainties of the fit parameters. A compromise between spatial resolution and the amount of noise in the incremental index-shift data has to be made.

During the first few seconds of heating, similar results are obtained for all methods for scanlines acquiring data from within the focus zone (Fig. 6). Discrete incremental index-shifts are sufficiently large for a sigmoid fit, which in turn is a good approximation for the data.

Significant differences between discrete and continuous calculations are found for the frame-to-frame methods (reference frames 4 s and 16 s before the examined frame) at all other times and in all other places: outside the focus zone even during the first seconds of heating (not shown), everywhere after the first seconds of heating (Fig. 7) and especially during cooling (Fig. 8). Discrete incremental index-shifts in these cases were very small and sometimes not even detectable (e.g. the detected index-shift is 0, the “true” index-shift is 0.4). For the frame-to-base-frame method, discrete calculation is possible for the whole heating and cooling process. The continuous index-shift serves as an additive correction and lowers uncertainty to some extent.

#### 1b. Influence of the reference frame on incremental index-shifts

The reference frame determines the absolute value of incremental index-shifts. They are larger, the earlier the reference was recorded: smallest for the frame-to-frame method with the reference 4 s before (Figs. 6 a, b, 7 a, b, and 8 a, b), larger for the reference 16 s before (Figs. 6 c, d, 7 c, d, and 8 c, d), and largest for the reference being the base frame (Figs. 6 e, f, 7 e, f, and 8 e, f). The incremental index-shift for a single interval is positive if the examined correlation interval was heated up or negative if it cooled down. Index-shifts which are too big (larger than six wavelengths, 96 indices in our calculation), which could arise for frame-to-base-frame methods, do not occur.

During cooling and when using a frame-to-frame method (Fig. 8 a–d), decreasing and negative incremental index-shifts are detectable within the former heated and then cooling zone. Rising shifts occur at the former heated zone’s edges (Fig. 8 b, d) because of heat transfer out of the heated zone to the borders of the phantom. The sigmoid function does not approximate this curve shape appropriately. Neither decreasing nor negative shifts occur in frame-to-base methods since the phantom at every scan heated up compared to the base frame.

In general, more noise exists in the incremental index-shift curves for the frame-to-base methods compared to the continuous frame-to-frame methods. This is due to a loss of coherence because of spatial extension and therefore decorrelation at higher temperatures. It mostly disappears again if temperatures fall below the threshold where the specific decorrelation occurred if no permanent structural changes arose. Structural changes from one frame to a frame recorded shortly before are few and small enough to be overcome by the algorithm. In addition, noise could, in general, be caused by the mechanical movement of the phantom but this is irrelevant under laboratory conditions. A frame-to-frame algorithm will be more stable against small mechanical movements as they occur, e.g. in living objects. Smaller intervals for incremental index-shift calculations lead to some extent to a better spatial resolution, larger ones to less noise in the calculated data.

#### 2. Temperature curves over time

**Fig. 9:**
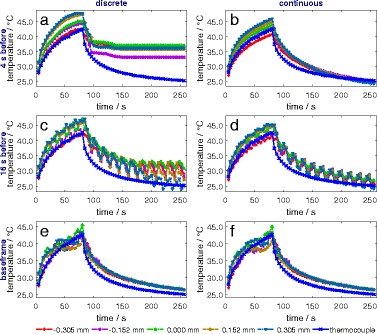
Calculated and measured temperatures over time (**a**–**f**). The frames 4 s and 16 s before the frame of interest correspond to the previous frame and the fourth frame before, respectively (80 s heating, *k*=747.9 °C, lateral neighbouring scanlines, uncertainty of the thermocouple 0.24 °C in maximum)

The temperature-time curves are derived from the sigmoid fit of the incremental index-shifts (Eq. 7) and shown in Fig. 9 along with independent measurements with a thermocouple. The lateral intervals, for which the temperature curves are shown, are very close to or identical to the thermocouple location and within the HIFU focus zone. The thermocouple location was determined by sight in the ultrasound base frame. The periodicity seen in Fig. 9 c, d is due to the chosen reference frame which is four frames (16 s) before the particular frame of interest.

Calculated temperature-time curves of neighbouring scanlines (lateral) and intervals (axial direction, not shown) are in good agreement with each other for the continuous methods and the discrete frame-to-base method. This agreement of calculated curves with each other is an indication of accuracy of the method, next to absolute calculated uncertainties. This is especially true of the lateral neighbouring scanlines since temperatures for every scanline are calculated with an independent sigmoid fit and no filtering in lateral direction was done. The maximum calculated temperatures are alike within the uncertainty.

For the frame-to-base methods (Fig. 9 e, f), calculated and measured temperatures are consistent within the uncertainty. An offset between calculated and thermocouple temperatures during cooling might be due to the evaporation of alcohol from the phantom during storage and measurement and therefore an effectively smaller value *k* than used for temperature calculation based on *k* measurement. This is very likely and inversely means that significantly higher temperatures occur in the thermocouple measurements during the second half of heating. On the other hand, higher temperatures are rather underestimated due to the method used (see “Temperature calculation with echo-time-shift and echo-index-shift” section). Both effects could eliminate each other. The remaining underestimation of calculated temperatures compared to thermocouple temperatures is due to the viscous heating artefact [47, 48].

For continuous frame-to-frame methods (Fig. 9 b, d), measured and calculated temperatures are consistent during heating. The cooling process is neither reproduced satisfyingly enough to be used for laboratory dosimetry nor for the validation of algorithms for treatment planning.

Discrete frame-to-frame methods reveal major shortcomings. These are due to very small and even falsely non-detected incremental index-shifts, large uncertainties of the sigmoid fit, and error propagation due to adding up of the calculated temperature differences over all frames. The discrete frame-to-frame methods are hence only suitable if a reference frame of some temporal distance is chosen.

#### 3. Two-dimensional temperature maps

**Fig. 10:**
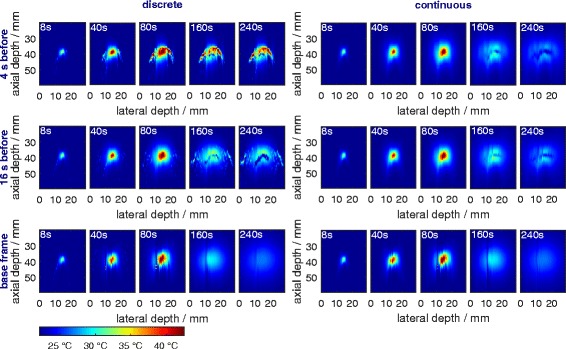
Temperature maps at different times. The phantom was actively heated with a HIFU transducer for 80 s and then cooled down for 180 s. The thermocouple reaches from the left-hand side up to 14 mm lateral depth into the phantom at an axial depth of 39 mm

Temperature maps are shown for all methods for different times during the heating and cooling process in Fig. 10. The location of the focus can be detected in all methods. The circular shape of the heated zone is displayed for the continuous and both frame-to-base methods but not for the discrete frame-to-frame methods. Noise on the left-hand side of the pictures is due to the thermocouple being located there.

The heating process can, in general, be seen for all methods, the cooling process only for the frame-to-base and continuous methods. The latter do not reproduce the circular shape nor a uniform temperature distribution during cooling.

### C. Uncertainty of temperature calculation

#### General thoughts on the uncertainty of temperature calculation

For the following uncertainty evaluation, the influence of *k* and the fit parameters (a to c) are considered. As mentioned before, spatial uncertainty is not taken into account separately since it strongly depends on measurement settings and can easily be added to the uncertainty budget. Depending on whether qualitative or quantitative temperature measurement is to be performed, uncertainties of different parameters have to be considered.

Qualitative temperature measurement is sufficient for determining the location of the focus, its shape and extension, or temperature distribution as percentiles. The uncertainty of *k* is irrelevant, whereas uncertainties of fit parameters and spatial resolution are crucial. The maximum acceptable spatial uncertainty depends on the size of the region to be treated and its closeness to risk organs. Percentiles of temperature distribution can be used to adjust the power of the therapeutic ultrasound device to meet treatment requirements (e.g. keep a minimum temperature in the focus zone for a certain time or stay below a maximum temperature at risk organs).

Quantitative temperature measurement resulting in the exact temperature-time profiles is, among other things, necessary for thermal dose calculation, to validate treatment plans and the algorithms to calculate them, to estimate bioeffects in the target region, and to prevent side effects in organs at risk.

The maximum acceptable uncertainty for calculated temperatures depends on the desired or suppressed effects and their onset temperature intervals. Since these temperatures are different for various types of tissue and dependent on other variables, too, no assessment about the absolute uncertainties will be given.

Among others, the following three prerequisites have to be fulfilled to properly apply uncertainty calculation as suggested in the *Guide to the Expression of Uncertainty in Measurement* (Section 2.3.4 in [44]):

- The first is that the fit model must be correct.
  The sigmoid fit model represents the incremental index-shift data reasonably for continuous methods, the discrete frame-to-base method, and the other discrete methods during the heating process within the heated zone. This is because the focus region is small and the transducer was not moved.
  Problems arise for frame-to-frame methods during cooling: shifts fall in the heated zone due to cooling and rise on its borders due to thermal conduction out of the heated zone (Fig. 8 b, d; heating and cooling occur along a scanline). The sigmoid model is also insufficient for discrete frame-to-frame methods since neither discretization of the underlying data nor the discretization error is taken into account for the fit. If the maximum incremental index-shift is only a few indices, incorrect steep or flat sigmoid fits result. These uncertainties do not exist for the continuous methods since any shift is continuously calculable.
  Depending on the type of measurement, other fit functions must be found, for instance, for validation of treatment plan algorithms with a moving transducer and multiple heated regions or if the focus is non-symmetric. Calculations can then be applied as shown in this paper.
- The second prerequisite is that variables have to be uncorrelated.
  Parameters *k*, *a*, *b*, and *c* have to be uncorrelated to each other (Eq. 7) for uncertainty calculation. Only *k* is independent. It has not been possible to express the other dependencies in mathematical terms up to now (Monte Carlo methods could be a solution). We assume that the correlation between fit parameters *a* and *b* cannot be neglected for uncertainty calculations and that both reinforce each other because of the turning point symmetry of the sigmoid curve. Correlations with fit parameter *d* can probably be neglected because its uncertainty is very small. Any correlation between *a* and *c* is probably very small, whereas *b* and *c* could influence each other.
- Filtered incremental index-shift data having to be representative is the third prerequisite.
  That means that filter algorithms should change or delete outliers in a representative way. Filtering is easiest for continuous frame-to-frame methods, quite challenging for frame-to-base methods and largely impossible for discrete frame-to-frame methods, because of small and discrete shifts. It might be laborious or not possible to correct the following effects: loss of correlation because of small mechanical movements, decorrelation due to thermal expansion and the thermoacoustic lens effect, signal instability of the ultrasound transducer, and change of chemical composition over time (i.e. due to healing of tissue or chemical instability of phantoms).
  Noise from the thermoacoustic lens’ effect could be reduced by methods like spatial compound imaging [28].

#### Uncertainty analysis of temperature calculation

The three prerequisites mentioned above are not fulfilled for the discrete variations. Therefore, only continuous temperature calculation variations are considered in the following.

Uncertainty of the sigmoid fit parameters was calculated with the residuals and Jacobian matrix (leads to 95 % intervals) and divided by two to obtain the standard deviation (67 % intervals). The uncertainty of *k* was determined in experiments performed by us.

**Fig. 11:**
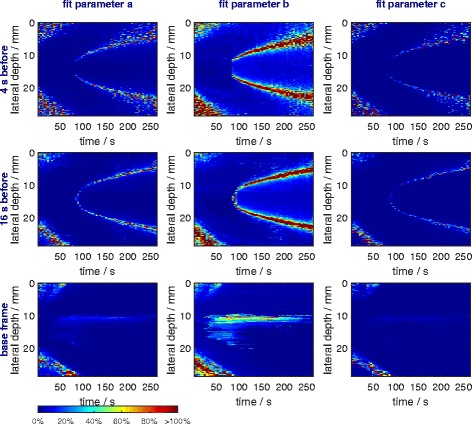
Uncertainties of the fit parameters. For every scanline (one pixel), a fit was performed and the obtained uncertainty (standard deviation) divided by the absolute value of the fit parameter of the associated scanline

In Fig. 11, uncertainties of the fit parameters *a*, *b*, and *c* are shown. They are larger in the outer region at the beginning of the heating process. This is because of small, incorrectly detected temperature changes due to noise in the data. Note that large relative uncertainties in these regions nevertheless lead to small or very small absolute temperature uncertainties due to the very small temperature change. The parabolic spreading of uncertainties for the frame-to-frame methods during cooling is due to incremental index-shift overshoots and the inability to reproduce this behaviour with a sigmoid fit (see “General thoughts on the uncertainty of temperature calculation” section). The impact is largest where a scanline meets the heated zones’ borders almost tangentially. Uncertainties in the frame-to-base method at the lateral depth of 10 mm are due to the thermocouple (Fig. 11). The thermocouple more likely influences incremental index-shift calculation for the frame-to-base method since large shifts and problems with decorrelation are intrinsic to the method. The thermoacoustic lens’ effect also leads to larger uncertainties and can be seen, for instance, in Fig. 10 for the frame-to-base method in the focus zone during the second half of the heating.

**Fig. 12:**
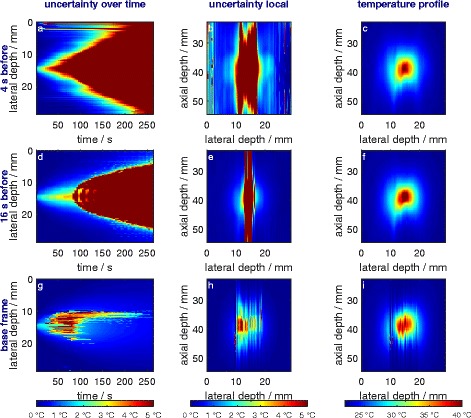
Absolute uncertainties and temperature changes are shown. *Left* (**a**, **d**, **g**): uncertainties over time in axial depth 38.7 mm (depth of focus zone, above thermocouple); *middle* (**b**, **e**, **h**): uncertainties at 88 s in both spatial directions; *right* (**c**, **f**, **i**): absolute temperature at 88 s

In Fig. 12, the absolute temperature uncertainties (Eq. 16) are shown for a plane through the focus zone. It should be mentioned again that uncertainties for the frame-to-frame methods add to the previous (4 s before) and fourth previous (16 s before) frames whereas uncertainties of the frame-to-base-frame method are calculated directly and do not add up. Uncertainties are calculated for every interval so that every calculated temperature value is associated with a calculation uncertainty. Our results suggest that temperature calculation can be significantly improved by developing better filter algorithms for incremental index-shifts, as well as by applying spatial and/or temporal filter algorithms to the final temperature maps.

For statistical evaluation (Table 2), we considered only the heated region where the calculated temperature changes were larger than 0.1 and 6.0 °C, respectively. Note that these uncertainties do not take all uncertainties due to spatial resolution into account. Spatial uncertainty will lead to an increase in uncertainty in regions of steep temperature gradients and strongly depend on the accuracy of the application and device settings. For these reasons, we did not take it into account.

**Table 2:** Quantiles of uncertainty values

	*δ𝜗*>0.1 °C			*δ𝜗*>6.0 °C		
	Q _50 %_ /°C	Q _5 %_ /°C	Q _95 %_ /°C	Q _50 %_ /°C	Q _5 %_ /°C	Q _95 %_ /°C
Heating and cooling						
Frame-to-frame 4 s before	3.92	0.20	377.30	24.77	1.57	389.81
Frame-to-frame 16 s before	0.47	0.05	79.10	8.06	0.97	112.44
Frame-to-base-frame	**0.11**	**0.02**	**0.97**	**1.26**	**0.78**	**4.73**
Only heating						
Frame-to-frame 4 s before	0.40	0.07	2.31	2.59	1.20	6.20
Frame-to-frame 16 s before	0.09	0.026	**1.01**	**1.33**	0.85	**2.59**
Frame-to-base-frame	**0.08**	**0.018**	2.02	2.04	**0.84**	5.74

Five percent, 50 % (median), and 95 % quantiles of absolute temperature uncertainty in the heated region (*δ𝜗*>0.1 °C and *δ𝜗*>6.0 °C) considering the heating and cooling process, as well as solely the heating process. Minimum values are in bold

#### Summary of uncertainty considerations

We investigated influences of the algorithmic evaluation (uncertainties of sigmoid fit parameters) as well as material properties (uncertainty of *k* which is due to the non-linear dependence of the speed of sound on temperature and thermal expansion). Depending on the purpose of the uncertainty consideration, frame-to-base methods or frame-to-frame methods, where the reference frame has a sufficiently large temporal distance to the frame of interest, are suitable. If heating and cooling processes are to be monitored, the reference frame generally has to have a larger temporal distance.

The main contribution to uncertainty results from *k*. It could be significantly lowered if the non-linearity were understood in more detail, if measurements were performed in other media or tissue where the relation of the speed of sound to temperature is more linear, or to a small amount if the measurement of *k* were enhanced. For tasks regarding human tissue, it is not possible to use different phantoms because the non-linear dependence of the speed of sound on temperature is inherent to non-fatty tissue.

Measuring errors are thought to be negligible compared to the uncertainties of the value *k*. The ‘true’ uncertainty might be larger than what is shown in Table 2, because correlations between fit parameters *a* and *b* are not taken into account and are not thought to be negligible.

## Conclusions

Discrete and continuous analysis variations of the echo-time-shift method with reference frames both 4 s and 16 s before the frame of interest along with the base frame were investigated. Special attention was paid to uncertainty considerations of the method in general and concrete measurements on a tissue-mimicking phantom.

The best results with regard to uncertainties, the reproducibility of the shape of the heated zone during heating and cooling, and in accordance with thermocouple measurements were obtained with the continuous frame-to-base-frame method. Continuous frame-to-frame methods provide good results during the heating process and additionally, a small robustness against small mechanical movements. Based on our findings, a minimum uncertainty of 12.4 % is intrinsic to the time-shift method within the range 20 °C to 44 °C due to tissue properties (non-linear dependence of the speed of sound on temperature). Algorithmic evaluation also adds to the uncertainty budget but with further processing (e.g. spatial and temporal filtering of temperature maps) and additional physical and biological assumptions it could significantly be reduced.

No uncertainty statement is possible for a single calculated temperature value. An uncertainty calculation was only reasonable for the continuous methods because of incalculable influences on uncertainties of the discrete methods. As far as possible, calculated temperatures were compared to and similar to thermocouple measurements.

Continuous time-shift calculations should be used not only for the quality assessment of therapeutic ultrasound devices but also for real-time therapy control because they outstandingly improve measurement compared to discrete methods. Discrete frame-to-frame methods have major shortcomings for laboratory dosimetry. The most significant ones are small incremental time-shifts from one frame to the reference frame and the additivity of uncertainties.

Methods using a reference frame a few seconds before the frame of interest are more robust against small mechanical movements and correlation loss due to thermal expansion and the change of interferences. Therefore, they seem most promising for various tasks.

Whether or not the achieved uncertainties are sufficient for laboratory dosimetry, validation of treatment plan algorithms, or decisions whether or not a therapeutic ultrasound device can be used for therapy, must be determined in further investigations. It is dependent on the biological responses of human tissue on different temperatures. To verify complex treatment plans, this method could be extended by altering or replacing the sigmoid fit function.

## Abbreviations

HIFU: High-intensity focused ultrasound
RF signal: Backscattered high-frequency signal
